# Genetic insights into 5-LOX-activating protein: a narrative review of disease associations

**DOI:** 10.1186/s40246-025-00867-x

**Published:** 2025-12-13

**Authors:** Katharina Rataj, Ulrike Garscha

**Affiliations:** Department of Pharmaceutical/Medicinal Chemistry, Institute of Pharmacy, Greifswald University, Friedrich-Ludwig-Jahn-Str. 17, 17489 Greifswald, Germany

**Keywords:** Polymorphism, 5-lipoxygenase-activating protein, Lipid mediator, Cardiovascular disease, Leukotrienes

## Abstract

The 5-lipoxygenase-activating protein (FLAP) is an integral membrane protein that is essential for 5-lipoxygenase-mediated leukotriene formation, thereby playing a key role in inflammation and serving as a potential therapeutic target. For over 30 years, researchers have been elucidating the crystal structure, identifying the inhibitor binding site, and developing several potent inhibitors, which have been investigated in preclinical and clinical studies to treat asthma, chronic kidney and cardiovascular diseases. However, despite being almost overlooked, more than 20,000 single nucleotide polymorphisms (SNPs) were detected in the *ALOX5AP* gene, coding for FLAP. To date, 66 SNPs have been studied in relation to a disease by different researchers, including different population groups. This review aims to synthesize current evidence on genetic variants of FLAP and delineate single SNPs that have been primarily implicated in coronary artery disease, myocardial infarction, and ischemic stroke. Associations between SNPs of *ALOX5AP* and these diseases have been reported, but findings remain inconsistent due to differences in study design, population diversity and methodological approaches. Although meta-analyses helped to integrate the results of different studies, they remain limited due to underlying differences and cannot provide a definitive conclusion.

## Introduction

Lipid mediators derived from polyunsaturated fatty acids are involved in inflammatory processes and are formed via different pathways, including cyclooxygenases (COX), lipoxygenases (LOX) and cytochrome P450 pathways [1–4]. Among the LOX pathways, the 5-LOX biosynthetic route is distinguished by the requirement for 5-LOX to translocate from the cytosol to the nuclear membrane, where it co-localizes with the integral membrane protein 5-LOX-activating protein (FLAP) [5–7]. This spatial recognition is essential for the full enzymatic activity of 5-LOX [8, 9] and enables the conversion of arachidonic acid (AA) into leukotrienes (LT), lipid mediators involved in the pathophysiology of inflammatory diseases such as asthma, atherosclerosis, arthritis and certain types of cancer [10].

Scientists at Merck discovered FLAP by elucidating the compound MK886 that potently inhibits cellular formation of LTs without interfering with 5-LOX [11]. FLAP could be successfully isolated as an 18 kDA protein using a radioactive MK886 photoaffinity analogue and a MK886 affinity gel [12]. The protein belongs to the MAPEG (membrane-associated proteins in eicosanoid and glutathione metabolism) superfamily, which includes the homologous enzymes LTC_4_-synthase and microsomal prostaglandin E synthase-1 [13]. Although these proteins have a conserved secondary structure, FLAP lacks its own enzymatic activity and cannot be modulated by glutathione due to the absence of a glutathione binding site. Radioligand binding and site-directed mutagenesis studies ultimately delineated the inhibitor binding pocket in FLAP, which partially overlaps with the AA binding site [14, 15]. Since then, FLAP has been described as a scaffold protein that acts as an AA transfer protein facilitating optimal substrate positioning and efficient dioxygenation by 5-LOX, forming the intermediate 5-hydroperoxyeicosatetraenoic acid (5-HPETE). Especially the following hydrogen abstraction by 5-LOX leading to LTA_4_ is promoted by FLAP [8]. However, the structural basis of the 5-LOX/FLAP complex formation remains unclear. On the 5-LOX side, it appears that four surface-exposed cysteine residues contribute to complex formation [16]. In contrast, our recent finding on the FLAP side indicates that the second cytosolic loop, particularly Ser108, plays a critical role in interacting with 5-LOX [17]. The crystal structure of FLAP complexed with MK591 revealed a cylindrical homotrimer. Each monomer consists of four transmembrane helices (α1-α4), connected by two relatively short cytosolic loops (C1 between α1 and α2, aa 38–41; C2 between α3 and α4, aa 105–115) and a luminal loop (L1 between α2 and α3, aa 78–80). In the FLAP homotrimer, three inhibitor-binding sites have been identified, each positioned at the interface between adjacent monomers within the membrane [18]. FLAP has emerged as an alternative therapeutic target to 5-LOX, and several inhibitors, such as fiboflapon (previously known as AM803 or GSK2190915) and atuliflapon (AZD5718), have been evaluated in clinical studies. However, no inhibitor has progressed beyond phase II [19–23].

So far, FLAP’s function has been primarily associated with lipid mediator formation, acting as an essential helper protein in leukotriene biosynthesis. In the last two decades, several genetic variants of the *ALOX5AP* gene, which encodes FLAP, have been investigated for their potential contribution to cardiovascular and cerebrovascular diseases, including coronary artery disease (CAD), myocardial infarction (MI), and ischemic stroke (IS). It is known that inflammatory processes contribute to the development of these diseases, which makes the involvement of FLAP conceivable [24–26]. However, FLAP´s role in the disease processes may also arise from another protein function that has yet to be identified. The aim of this review is to present an overview of the investigations that have been conducted to link certain genetic variants to these diseases, and to summarize the reported associations with respect to different populations.

## Characterization of the *ALOX5AP* gene encoding FLAP

**Fig. 1:**
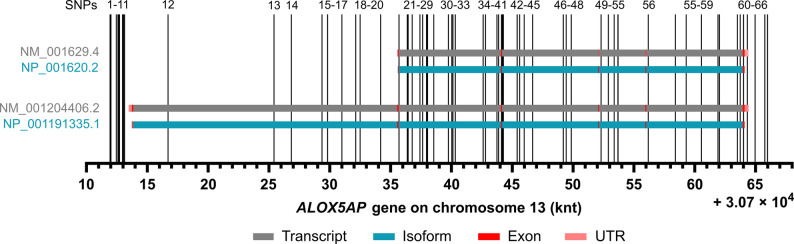
Single nucleotide polymorphisms (SNPs) within the *ALOX5AP* gene encoding FLAP. SNPs 1-66 were selected based on their association with diseases. *ALOX5AP* contains two transcript variants NM_001629.4 and NM_001204406.2 (grey bars) encoding the isoforms NP_001620.2 and NP_001191335.1 (blue bars). The exons (red) and UTRs (= untranslated region, light red) are marked within this.

FLAP is encoded by the *ALOX5AP* gene, which was mapped to the 13q12 region on chromosome 13 using radiation hybrids, inclusion in mapped clones and in situ hybridization [27]. There are three transcript variants for this gene available, of which NM_001629.4 (transcript variant 1) is the predominant form, encoding for the 161 aa protein variant NP_001620.2 (isoform 1) (Fig. 1). In 1991 Kennedy et al*.* proposed the structure of this transcript variant, which spans > 31 kbp and consists of five small exons and four large introns [28]. The first four exons range in size from 71 to 144 bp. The final exon is the largest, measuring 478 bp [28]. The transcript variant NM_001204406.2 (transcript variant 2) has an alternate 5´ sequence, which includes an upstream in-frame AUG start codon and an additional exon, compared to variant 1 (provided by RefSeq from NCBI). The resulting protein variant NP_001191335.1 (isoform 2) is 218 amino acids long and has an extended N-terminus compared to isoform 1. Transcript variant 3 (XM-017020522.3) is not discussed in this review because no reference is available.

## Single-nucleotide polymorphisms in *ALOX5AP*

Single-nucleotide polymorphisms (SNPs) are DNA sequence variations that arise from a change in a single nucleotide base. They are heritable and commonly shared among individuals of the same ancestry, and need to be distinguished from spontaneously occurring mutations. By definition, a nucleotide variation is only classified as a SNP if it is present in at least 1 % of a given population [29]. SNPs are the most common type of genetic variation with a frequency of about 0.1 % across the genome, and are being studied for the onset of various diseases [29, 30]. They can occur in both the non-coding and coding regions of a gene, with the latter being divided into missense SNPs, which change the encoded amino acid, and synonymous (silent) SNPs [30]. Non-coding regions include introns and the areas before and after the gene, and contain important structural motifs such as enhancer elements, DNase hypersensitivity regions and chromatin marks that are required for the regulation of gene expression [31]. As a result, most of the SNPs that provide statistical evidence of an increased risk for complex diseases are associated with these regions [31].

More than 20,000 SNPs have been identified in the *ALOX5AP* gene (provided by the SNP database from NCBI). For this review, however, we limited our analysis to SNPs with PubMed-indexed evidence of disease association, narrowing the set to 66 SNPs Fig. 1. Table 1 summarizes the 66 SNPs, their allelic variants, the associated diseases investigated, as well as the population groups included in the respective studies.

**Table 1:** Overview of the selected SNPs within *ALOX5AP*

No	SNP	Position (nt)	Alleles	Association	Study Subjects	Pub
1	rs34536374	30,711,952	C > T	No association with IS	Chinese populations, 200 cases/200 controls and 810 cases/825 controls	[37]
2	rs34344566	30,711,954	A > C,G	No association with IS	Chinese populations,200 cases/200 controls and 810 cases/825 controls	[37]
3	rs34352240	30,711,963	C > G,T	No association with IS	Chinese populations,200 cases/200 controls and 810 cases/825 controls	[37]
4	rs9578194	30,712,013	G > A,C,T	No association with IS	Chinese populations,200 cases/200 controls and 810 cases/825 controls	[37]
5	rs55950839	30,712,463	T > A	No association with IS	Chinese populations,200 cases/200 controls and 810 cases/825 controls	[37]
6	rs55780307	30,712,598	A > G,T	No association with IS	Chinese populations,200 cases/200 controls and 810 cases/825 controls	[37]
7	rs59227506	30,712,649	T > C	No association with IS	Chinese populations,200 cases/200 controls and 810 cases/825 controls	[37]
8	rs34404999	30,712,718	C > A,T	No association with IS	Chinese populations,200 cases/200 controls and 810 cases/825 controls	[37]
9	rs12560847	30,712,965	C > A,G,T	No association with IS	Chinese populations,200 cases/200 controls and 810 cases/825 controls	[37]
10	rs9578195	30,713,053	G > A	No association with IS	Chinese populations,200 cases/200 controls and 810 cases/825 controls	[37]
11	rs61947373	30,713,131	G > A	No association with IS	Chinese populations,200 cases/200 controls and 810 cases/825 controls	[37]
12	rs9669952	30,716,712	T > C	**Negative association of C allele with T2DM**	**Elderly** **Greek population**, **716 cases/569 controls**	[100]
13	rs17222814	30,725,416	G > A	**Association of HapA with EOCAD in Caucasian sample; no association in Americans and overall test**	**Caucasian and American population, 656 cases/405 controls**	[54]
				No association of HapA with CAD	Caucasian population, 2,834 cases/913 controls African American population, 502 cases/263 controls	[39]
				No association of HapA with CAD	European-American population, 500 cases/500 controls	[52]
				**Association of HapA with MI** No association of rs17222814 with CAD	**Meta-analysis of 13 studies of 11 Caucasian and 2 Asian populations** **5,398 cases in total /4,937 controls in total for HapA** 6,772 cases in total /3,478 controls in total for rs17222814	[48]
				No association with carotid artery intima-media thickness (IMT)	German population, 969 and 1,905 subjects	[40]
				No significant association with carotid artery intima-media thickness (IMT) as marker for carotid atherosclerosis	Young Finn population, 813 subjects	[41]
				**Protective for ISR**	**US population, 46 cases/46 controls**	[42]
				No association with MI	Danish population, 2,876 cases/2,858 controls	[43]
				No association of HapA with MI	German population, 1,211 cases/1,015 controls	[49]
				No association with IS of single SNV and HapA	Meta-analysis of 6 studies of 4 Caucasian and 2 Asian populations	[38]
				No association of HapA with IS	Swedish population, 685 cases/751 controls	[50]
				No association of HapA with IS	Northern Chinese Han population, 682 cases/598 controls	[51]
				No association with stroke	Taiwanese population, 291 cases/278 controls	[53]
				No association of HapA with brain infarcts defined by Magnetic Resonance Imaging (MRI)	Caribbean Hispanic population, 202 cases/487 controls European population, 1,823 cases/7,578 controls	[72]
				No significant association with SSc	European Caucasian population, 977 cases/558 controls	[45]
				No association with depression	US population, 1,368 subjects	[46]
				No significant association with xanthomas in hypercholesterolemia	Dutch population, 541 cases/494 controls	[47]
14	rs12430915	30,726,842	T > C	No significant association with chronic rhinosinusitis	Canadian (~ 90 % White) population, 206 cases/200 controls	[59]
15	rs4769870	30,729,374	C > T	No significant association with chronic rhinosinusitis	Canadian (~ 90 % White) population, 206 cases/200 controls	[59]
16	rs17216473	30,729,828	G > A,T	**Significant association of A allele with risk of MI and G allele with absence of MI**	**Ukrainian population, 95 cases/110 controls**	[56]
				**Association of HapB with increased risk of MI**	**German population, 1,211 cases/1,015 controls**	[49]
				**Association with increased risk of ISR**	**US population, 46 cases/46 controls**	[42]
				No association with risk of coronary ISR	Czech population, 160 cases/160 controls	[57]
				**Significant association of HapB** (but not rs17216473 as single SNP) **with CAD**	**Meta-analysis of 13 studies of 11 Caucasian and 2 Asian populations,** **4,619 cases in total/4,313 controls in total for HapB** 7,106 cases in total/5,326controls in total for rs17216473	[48]
				**Significant association of rs17216473 and HapB with risk of premature CAD**	**European-American population, 500 cases/500 controls**	[52]
				No association of HapB with EOCAD or MI	Caucasian and American population, 656 cases/405 controls	[54]
				No association of HapB with IS	Meta-analysis of 4 studies of 3 Caucasian and 1 Asian population	[38]
				No association with depression	US population, 1,368 subjects	[46]
				No significant association with SSc and -related interstitial lung disease	European Caucasian population, 977 cases/558 controls	[45]
				No significant association with xanthomas in hypercholesterolemia	Dutch population, 541 cases/494 controls	[47]
17	rs4076128	30,731,006	A > G	**Association with reduced risk of thyroid cancer**	**Chinese population, 520 cases/520 controls**	[101]
				No significant association with breast cancer risk across race or ethnicity	Latina, African-American, White woman populations, 802 cases in total/888 controls in total	[60]
				No significant association with chronic rhinosinusitis	Canadian (~ 90 % White) population, 206 cases/200 controls	[59]
18	rs4073259	30,732,134	G > A	**Significant association with CI; Positive association of A allele and negative association of GG genotype**	**Chinese Han population, 236 cases/219 controls**	[44]
				**Significant association with IS; G allele and GG genotype frequency significantly lower in IS cases**	**Chinese Henan Han population, 150 cases/155 controls and 400 cases/ 400 controls**	[62]
				No significant association with IS	Northeastern Chinese Han population, 501 cases/497 controls	[63]
				No significant association with IS	Northeastern Chinese Han population, 507 cases/503controls	[102]
				No association with IS	Meta-analysis of 4 studies of 4 Asian populations	[38]
				No association with MI	Danish population, 2,876 cases/2,858 controls	[43]
				No association with thyroid cancer	Chinese population, 520 cases/520 controls	[101]
				No significant association with breast cancer risk across race or ethnicity	Latina, African-American, White woman populations, 802 cases in total/888 controls in total	[60, 61]
				No association with prostate cancer	Non-Hispanic white population, 585 cases/585 controls	[61]
19	rs11616333	30,732,492	C > G	No significant association with chronic rhinosinusitis	Canadian (~ 90% White) population, 206 cases/200 controls	[59]
20	rs17222919	30,734,192	T > A,G	**Significant association with IS; Risk of developing IS decreased in presence of T allele and increased in presence of G allele**	**Iranian population, 228 cases/200 controls**	[64]
				**Significant Association with stroke; Sensitivity analysis indicated instability of results**	**Meta-analysis of 5 studies of Chinese and Korean populations,** **4,120 cases in total/4,372 controls in total**	[66]
				**Significant association with IS; G allele frequency was significantly lower in IS group**	**Chinese populations,** **200 cases/200 controls and 810 cases/825 controls**	[37]
				**Significant association with IS; G allele frequency was significantly lower in IS group**	**Chinese Han populations,** **910 cases/925 controls and 1,003 cases/889 controls**	[67]
				**Significant association of TG genotype with increased risk of IS**	**Chinese population, 658 cases/704 controls**	[68]
				No association with IS	Meta-analysis of 4 studies of 4 Asian populations	[38]
				**Association with intracerebral hemorrhage, but not IS**	**Korean population, 79 cases/398 controls**	[38, 103]
21	rs4769055	30,735,693	C > A	**Significant association with IS; AA genotype were significantly higher in controls than IS group**	**Greek population, 213 cases/210 controls**	[104]
				**Association with prevalence of atherothrombotic CI in patients with metabolic syndrome**	**Japanese population, 313 cases/971 controls**	[105]
				No significant association with atherosclerosis	Caucasian population, 828 cases/170 controls	[90]
				No association with CAD	US population, 1,552 cases/1,583 controls	[84]
				No significant association with chronic rhinosinusitis	Canadian (~ 90 % White) population, 206 cases/200 controls	[59]
22	rs9579645	30,736,369	A > C	**Association of interaction with glyphosate and prostate cancer risk**	**97 % White population, 776 cases/1,444 controls**	[106]
				No association with cardiovascular disease in HIV patients	Spanish Caucasian population, 83 cases/130 controls	[70]
23	rs9579646	30,736,442	G > A,C,T	**Significant association with IS**	**US population (Greater Cincinnati/Northern Kentucky),** **357 cases/482 controls**	[69]
				**Significant association of GG genotype with increased risk of CI**	**Chinese Han population, 236 cases/219 controls**	[44]
				**Significant association of G allele frequency and haplotype composed of rs9579646 and rs10507391 (GT and GA) with increased risk of CI**	**Northeastern Chinese Han population, 456 cases/452 controls**	[80, 81]
				**Significant association of AG genotype with decreased risk of stroke compared with AA genotype**	**Eastern Chinese Han population, 507 cases/510 controls**	[80]
				No significant association with IS	Chinese populations, 200 cases/200 controls and 810 cases/825 controls	[37]
				**Significant association of TG genotype with increased risk of IS**	**Chinese population, 658 cases/704 controls**	[68]
				**Significant association with decreased risk of CAD**	**Lebanese population, 1,370 cases, 589 control**s	[25]
				No association with CAD	Meta-analysis of 13 studies of 11 Caucasian and 2 Asian populations 3,998 cases in total /1,552 controls in total	[48]
				No association with cardiovascular disease in HIV patients	Spanish Caucasian population, 83 cases/130 controls	[70]
24	rs4075131	30,736,782	A > G	No association with cardiovascular disease in HIV patients	Spanish Caucasian population, 83 cases/130 controls	[70]
25	rs9578196	30,737,420	C > T	**Association with cardiovascular disease in HIV patients**	**Spanish Caucasian population, 83 cases/130 controls**	[70]
				No significant association with chronic rhinosinusitis	Canadian (~ 90 % White) population, 206 cases/200 controls	[59]
26	rs4293222	30,737,636	C > A,G,T	**Positive significant association of three-way combination of rs4293222, rs10900213 (ALOX5) and rs2107545 (MPO) with risk of IS**	**Chinese Han population, 351 cases/417 controls**	[107]
				**Significant association with atherothrombotic stroke (ATS); GG and CG genotype associated with increased risk compared to CC genotype**	**Taiwanese population, 291 cases/278 controls**	[53]
				No association with cardiovascular disease in HIV patients	Spanish Caucasian population, 83 cases/130 controls	[70]
				No significant association with risk of colon and rectal cancer stratified by fat and fatty acid intake	US population (Utah, Northern California, Minnesota), 1,543 cases/1,900 controls (Colon) and 712 cases/912 controls (Rectum)	[108]
				No association with T2DM	Chinese population, 396 cases/678 controls	[109]
				No significant association with chronic rhinosinusitis	Canadian (~ 90 % White) population, 206 cases/200 controls	[59]
27	rs10507391	30,737,959	A > C,T	**Significant association with IS in Caucasians from Europe, but not from other countries**	**Meta-analysis of 9 studies of US, European, Spanish, Portuguese, Russian and Moroccan** **Caucasian populations,** **4,198 cases/3,699 controls in total**	[77]
				**Significant association of T allele with increased risk of IS**	**Iberian population, 1,092 cases/781 controls**	[78]
				**Significant association of haplotype composed of rs10507391 and rs12429692 with decreased risk of stroke**	**Eastern Chinese Han population, 507 cases/510 controls**	[80]
				**Association of interaction of rs10507391 and rs776746 (CYP3A5) with increased risk of CI**	**Chinese population, 292 cases/259 controls**	[26]
				**Significant association of interaction of rs10507391 AA and rs776746 (CYP3A5) GG with increased risk of IS**	**Chinese population, 396 cases/378 controls**	[82]
				**Significant association of Haplotype composed of rs9579646 and rs10507391 (GT and GA) with increased risk of CI**	**Northeastern Chinese Han population, 456 cases/452 controls**	[81]
				**Association of TT/TA genotype with increased risk of CI (additionally through smoking)**	**Northern Chinese Han population, 420 cases/488 controls**	[79]
				**Significant association of rs10507391 together with rs20417 (COX-2) with increased risk for CI**	**Chinese population 411 cases/411 controls**	[83]
				No association with IS of rs10507391, HapA and HapB	Meta-analysis of 20 studies of 6 Caucasian and 14 Asian populations (rs10507391), 4 Caucasian populations (HapA), 3 Caucasian and 1 Asian population (HapB)	[38]
				No significant association with IS in female patients	Chinese Han population, 459 cases/462 controls	[76]
				No association of HapA with IS	Swedish population, 685 cases/751 controls	[50]
				**Significant association of AA genotype and A allele** (but not of HapA) **with IS**	**Northern Chinese Han population, 682 cases/598 controls**	[51]
				No association with risk of CI	Meta-analysis of 12 studies of Chinese populations, 6,844 cases/7,850 controls in total	[71]
				No significant association with CI	Chinese Han population, 236 cases/219 controls	[44]
				No association with CI	Chinese Han population, 547 cases/794 controls	[75]
				No significant association with IS	Chinese Han population 690 cases/767 controls	[73]
				No significant association with acute stroke development	Russian population, 1,320 cases/467 controls	[74]
				No association with stroke	Taiwanese population, 291 cases/278 controls	[53]
				No association of HapA with brain infarcts defined by Magnetic Resonance Imaging (MRI)	Caribbean Hispanic population, 202 cases/487 controls European population, 1,823 cases/7,578 controls	[72]
				**Significant association of HapA** (but not HapB) **with EOCAD in Caucasians; no association in americans and overall test**	**Caucasian and American population, 656 cases/405 controls**	[54]
				**Significant association of** **HapB** **(**but not rs10507391 as single SNP**)** **with** **CAD** **Association of HapA with MI**	**Meta-analysis of 13 studies of 11 Caucasian and 2 Asian populations,** **4,619 cases in total/4,313 controls in total for HapB** 7,407cases in total /5,432 in total controls for rs10507391 **5,398 cases in total /4,937 controls in total for HapA**	[48]
				**Association of HapB** (but not HapA) **with increased risk of MI**	**German population, 1,211 cases/1,015 controls**	[49]
				**Significant association of HapB** (but not HapA) **with risk of premature CAD**	**European-American population, 500 cases/500 controls**	[52]
				No association with CAD	US population, 1,552 cases/1,583 controls	[84]
				No association of HapA with CAD	Caucasian population, 2,834 cases/913 controls African American population, 502 cases/263 controls	[39]
				No association with acute coronary syndrome	Chinese Han population, 508 cases/201 controls	[92]
				**Associated with increased risk of ISR; HapA protective against restenosis**	**US population, 46 cases/46 controls**	[42]
				**Significant association of A allele in interaction with rs17525488 (LTA4H) with decreased risk of asthma and lower baseline lung function**	**Mexican and Puerto Rican population, 296 and 391 families**	[87]
				**Association with augmentation of bronchodilator responsiveness with leukotriene modifier in occurrence of minor allele rs2540491 (LTA4H) or major allele rs2540487 (LTA4H)**	**Mexican and Puerto Rican population, 293 and 356 subjects**	[88]
				**Association of A allele with increased risk of asthma**	**Caucasian population, 341 families**	[85]
				**Association with increased risk of asthma**	**Chinese Han population, 60 cases/61 controls**	[86]
				**Association with risk of SSc and -related interstitial lung disease; A allele is associated with increased synthesis of cysteinyl leukotrienes**	**European Caucasian population, 977 cases/558 controls**	[45]
				**Association of AA genotype with decreased risk of myeloid leukemia**	**Chinese population, 150 cases/134 controls**	[89]
				No association with MI, but association with total cholesterol levels	Chinese Han population, 401 cases/409 controls	[24]
				No association with MI	Danish population, 2,876 cases/2,858 controls	[43]
				No significant association with atherosclerosis	Caucasian population, 828 cases/170 controls	[90]
				No association with depression	US population, 1,368 subjects	[46]
				No association with AD	Czech population, 335 cases/378 controls	[91]
				No significant association with xanthomas in hypercholesterolemia	Dutch population, 541 cases/494 controls	[47]
28	rs12429692	30,738,041	A > C,T	**Significant association of haplotype composed of rs10507391 and rs12429692 with decreased risk of stroke**	**Eastern Chinese Han population, 507 cases/510 controls**	[80]
				No significant association with risk of IS	Eastern Chinese Han population, 690 cases/767 controls	[73, 110]
				No significant association with atherothrombotic stroke (ATS)	Taiwanese population, 291 cases/278 controls	[53]
				**Significant association with increased risk of adenoma**	**US population (Minnesota), 485 cases/578 controls**	[111]
				No association with cardiovascular disease in HIV patients	Spanish Caucasian population, 83 cases/130 controls	[70]
				No significant association with chronic rhinosinusitis	Canadian (~ 90 % White) population, 206 cases/200 controls	[59]
29	rs4769873	30,738,552	C > T	**Association with cardiovascular disease in HIV patients**	**Spanish Caucasian population, 83 cases/130 controls**	[70]
30	rs9315045	30,739,752	T > A,C,G	No association with cardiovascular disease in HIV patients	Spanish Caucasian population, 83 cases/130 controls	[70]
31	rs4503649	30,740,037	G > A,C	No association with cardiovascular disease in HIV patients	Spanish Caucasian population, 83 cases/130 controls	[70]
				No significant association with chronic rhinosinusitis	Canadian (~ 90 % White) population, 206 cases/200 controls	[59]
32	rs9508832	30,740,127	G > A	**Association of regular NSAID users with variant genotype with additional decreased risk of rectal cancer**	**US population (Minnesota), 485 cases/578 controls**	[111]
33	rs3885907	30,740,318	C > A,G,T	**Association with cardiovascular disease in HIV patients**	**Spanish Caucasian population, 83 cases/130 controls**	[70]
				**Association with chemotherapy-induced alopecia in patients treated with cyclophosphamide + epirubicin + / − 5-FU (CEF)**	**Japanese population, 303 cases/880 controls**	[112]
34	rs10162089	30,742,601	G > A	**Significant association of A allele with Forced expiratory volume in one second (FEV1) (marker for lung function)**	**Korean population, 3,627 subjects**	[113]
				No association with cardiovascular disease in HIV patients	Spanish Caucasian population, 83 cases/130 controls	[70]
				No significant association with chronic rhinosinusitis	Canadian (~ 90 % White) population, 206 cases/200 controls	[59]
35	rs9551960	30,742,803	G > A,C,T	**Association with NSAID use and rectal cancer**	**US population (Minnesota), 485 cases/578 controls**	[111]
				No significant association with atherosclerosis	Caucasian population, 828 cases/170 controls	[90]
36	rs4254165	30,743,741	A > G	No association with cardiovascular disease in HIV patients	Spanish Caucasian population, 83 cases/130 controls	[70]
				No significant association with chronic rhinosinusitis	Canadian (~ 90 % White) population, 206 cases/200 controls	[59]
37	rs4360791	30,743,883	G > A	**Association of AA and AG genotype with increased risk of atherothrombotic stroke (ATS) compared with GG genotype**	**Taiwanese population, 291 cases/278 controls**	[53]
38	rs41351946	30,744,112	C > G,T	**Association with T2DM**	**Polish population, 303 subjects**	[93]
39	rs3803277	30,744,171	C > A,G	**Significant association of CA genotype with decreased risk of stroke**	**Greek population, 213 cases/210 controls**	[104]
				**Significant association of A allele with Forced expiratory volume in one second (FEV1) (marker for lung function)**	**Korean population, 3,627 subjects**	[113]
				**Some evidence for association with asthma**	**Caucasian population, 1,877 subjects**	[114]
				No association with CAD	US population, 1,552 cases/1,583 controls	[84]
				No significant association with baseline lung function or COPD susceptibility in smokers	UK population, 599 cases/176 controls	[115]
				No significant association with CAD	US population, 1,155 cases/730 controls	[116]
40	rs202068154	30,744,203	A > C,G	**Significant association of AC genotype and decreased risk of stroke**	**Greek population, 213 cases/210 controls**	[104, 116]
41	rs3803278	30,744,264	T > A,C	**Significant positive association of C allele with diabetes nephropathy in patients with T2DM**	**Slovenian Caucasian population,** **276 cases/375 controls**	[117]
				**Significant association of C allele and decreased risk of chronic kidney disease (CKD) in individuals with T2DM**	**Japanese population, 636 cases/1,106 controls**	[118]
				**Significant association with MI**	Japanese population, 353 cases/1,875 controls	[119]
				No association with CAD	US population, 1,552 cases/1,583 controls	[84]
				No significant association with risk of colon and rectal cancer stratified by fat and fatty acid intake	US population (Utah, Northern California, Minnesota), 1,543 cases/1,900 controls (Colon) and 712 cases/912 controls (Rectum)	[108]
42	rs4356336	30,745,409	T > C,G	No significant association with chronic rhinosinusitis	Canadian (~ 90 % White) population, 206 cases/200 controls	[59]
43	rs17238773	30,745,614	A > T	No significant association with chronic rhinosinusitis	Canadian (~ 90 % White) population, 206 cases/200 controls	[59]
44	rs4147064	30,745,981	T > A,C,G	**Association of CT genotype with CI in patients with hypertension**	**Chinese Han population, 236 cases/219 controls**	[44]
				No association with IS	Northeastern Chinese Han population, 456 cases/452 controls	[81]
				No significant association with CAD	US population, 1,155 cases/730 controls	[116]
45	rs9506352	30,746,678	G > A	**Significant association of A allele with Forced expiratory volume in one second (FEV1) (marker for lung function)**	**Korean population, 3,627 subjects**	[113]
				No significant association with CAD	US population, 1,155 cases/730 controls	[116]
				No significant association with atherosclerosis	Caucasian population, 828 cases/170 controls	[90]
				No significant association with risk of colon and rectal cancer stratified by fat and fatty acid intake	US population (Utah, Northern California, Minnesota), 1,543 cases/1,900 controls (Colon) and 712 cases/912 controls (Rectum)	[108]
				No significant association with chronic rhinosinusitis	Canadian (~ 90 % White) population, 206 cases/200 controls	[59]
46	rs4075692	30,749,205	A > G,T	**Association of regular NSAID users with variant genotype with additional decreased risk of rectal cancer**	**US population (Minnesota), 485 cases/578 controls**	[111]
47	rs17245197	30,749,435	C > A,G,T	No significant association with risk of colon and rectal cancer stratified by fat and fatty acid intake	US population (Utah, Northern California, Minnesota), 1,543 cases/1,900 controls (Colon) and 712 cases/912 controls (Rectum)	[108]
48	rs17245204	30,749,851	C > T	No association with cardiovascular disease in HIV patients	Spanish Caucasian population, 83 cases/130 controls	[70]
49	rs4769874	30,752,304	G > A	**Significant association with IS**	**US population (Greater Cincinnati/Northern Kentucky),** **357 cases/482 controls**	[69]
				No significant association with IS in female patients	Chinese Han population, 459 cases/462 controls	[76]
				No association with IS of rs4769874 and HapA	Meta-analysis of 13 studies of 4 Caucasian and 9 Asian populations	[38]
				No association of HapA with IS	Northern Chinese Han population, 682 cases/598 controls	[51]
				No significant association with IS	Chinese population, 396 cases/378 controls	[82]
				No significant association with CI	Chinese population, 292 cases/259 controls	[26]
				No significant association with CI	Chinese Han population, 236 cases/219 controls	[44]
				No association with CI	Northern Chinese Han population, 420 cases/488 controls	[79]
				No association with IS	Northeastern Chinese Han population, 456 cases/452 controls	[81]
				No association with stroke	Taiwanese population, 291 cases/278 controls	[53]
				No association of HapA with brain infarcts defined by Magnetic Resonance Imaging (MRI)	Caribbean Hispanic population, 202 cases/487 controls European population, 1,823 cases/7,578 controls	[72]
				No association of HapA with IS	Swedish population, 685 cases/751 controls	[50]
				**Significant association with increased risk of CAD**	**Lebanese population, 1,370 cases/589 controls**	[25]
				**Significant association of HapA with EOCAD in Caucasians; no association in Americans and overall test**	**Caucasian and American population, 656 cases/405 controls**	[54]
				No association of HapA with CAD	Caucasian population, 2,834 cases/913 controls African American population, 502 cases/263 controls	[39]
				No association of HapA with CAD	European-American population, 500 cases/500 controls	[52]
				**Association of HapA with MI** No association of rs4769874 with CAD	**Meta-analysis of 13 studies of 11 Caucasian and 2 Asian populations** **5,398 cases in total /4,937 controls in total for HapA** 7,388 cases in total /5,405 controls in total for rs4769874	[48]
				No association with acute coronary syndrome	Chinese Han population, 508 cases/201 controls	[92]
				No significant association with atherosclerosis	Caucasian population, 828 cases/170 controls	[90]
				No association with MI	Danish population, 2,876 cases/2,858 controls	[43]
				No association of HapA with MI	German population, 1,211 cases/1,015 controls	[49]
				**Significant association of A allele with increased risk of AD**	**Czech population, 335 cases/378 controls**	[91]
				No association with depression	US population, 1,368 subjects	[46]
				No association with myeloid leukemia	Chinese population, 150 cases/134 controls	[89]
				No significant association with SSc and -related internal organ involvement	European Caucasian population, 977 cases/558 controls	[45]
				No significant association with xanthomas in hypercholesterolemia	Dutch population, 541 cases/494 controls	[47]
50	rs9579648	30,752,895	G > C,T	No association with cardiovascular disease in HIV patients	Spanish Caucasian population, 83 cases/130 controls	[70]
				No significant association with chronic rhinosinusitis	Canadian (~ 90 % White) population, 206 cases/200 controls	[59]
51	rs10507393	30,753,384	T > C,G	No association with cardiovascular disease in HIV patients	Spanish Caucasian population, 83 cases/130 controls	[70]
52	rs9579649	30,753,685	C > T	No significant association with chronic rhinosinusitis	Canadian (~ 90 % White) population, 206 cases/200 controls	[59]
53	rs9315048	30,753,703	G > A,C,T	No association with cardiovascular disease in HIV patients	Spanish Caucasian population, 83 cases/130 controls	[70]
				No significant association with atherosclerosis	Caucasian population, 828 cases/170 controls	[90]
54	rs4468448	30,756,179	T > A,C,G	**Some evidence for association with asthma, but did not survive correction for multiple testing**	**Caucasian population, 1,877 subjects**	[114]
				No significant association with baseline lung function or COPD susceptibility in smokers	UK population, 599 cases/176 controls	[115]
				No significant Association with Sickle Cell Diseases	US population, 1,518 and 211 subjects	[120]
55	rs9551963	30,758,410	A > C,T	**Significant association of AC genotype with increased risk of CI in patients with hypertension**	**Chinese Han population, 236 cases/219 controls**	[44]
				**Association of CC/CA genotype with increased risk of CI under smoking**	**Northern Chinese Han population, 420 cases/488 controls**	[79]
				**Significant association with IS in female patients**	**Chinese Han population, 459 cases/462 controls**	[76]
				No association with IS of rs9551963 and HapA	Meta-analysis of 12 studies of 5 Caucasian and 7 Asian populations (rs9551963), 4 Caucasian populations (HapA)	[38]
				No association of HapA with IS	Swedish population, 685 cases/751 controls	[50]
				No association of HapA with IS	Northern Chinese Han population, 682 cases/598 controls	[51]
				No association with IS	Chinese population, 396 cases/378 controls	[82]
				No significant association with CI	Chinese population, 292 cases/259 controls	[26]
				No association with CI	Chinese Han population, 547 cases/794 controls	[75]
				No association with IS	Northeastern Chinese Han population, 456 cases/452 controls	[81]
				No significant association with acute stroke development	Russian population, 1,320 cases/467 controls	[74]
				No association with stroke	Taiwanese population, 291 cases/278 controls	[53]
				No association of HapA with brain infarcts defined by Magnetic Resonance Imaging (MRI)	Caribbean Hispanic population, 202 cases/487 controls European population, 1,823 cases/7,578 controls	[72]
				**Significant association of HapA with EOCAD in Caucasians; no association in Americans and overall test**	**Caucasian and American population, 656 cases/405 controls**	[54]
				**Association of HapA with MI** No association of rs9551963 with CAD	**Meta-analysis of 13 studies of 11 Caucasian and 2 Asian populations** **5,398 cases in total /4,937 controls in total for HapA** 7,125 cases in total /5,353 controls in total for rs9551963	[48]
				No association of HapA with MI	German population, 1,211 cases/1,015 controls	[49]
				No association of HapA with CAD	Caucasian population, 2,834 cases/913 controls African American population, 502 cases/263 controls	[39]
				No association of HapA with CAD	European-American population, 500 cases/500 controls	[52]
				**Significant association with decreased risk of MI in men**	**Danish population, 2,876 cases/2,858 controls**	[43]
				**Association with augmentation of bronchodilator responsiveness with leukotriene modifier in occurrence of minor allele rs2540491 (LTA4H) or major allele rs2540487 (LTA4H)**	**Mexican and Puerto Rican population, 293 and 356 subjects**	[88]
				No association with asthma	Mexican and Puerto Rican population, 296 and 391 families	[87]
				**Significant association of A allele with increased risk of xanthomas in hypercholesterolemia compared with CC genotype**	**Dutch population, 541 cases/494 controls**	[47]
				No significant association with carotid artery intima-media thickness (IMT) as marker for carotid atherosclerosis	Young Finn population, 813 subjects	[41]
				No association with depression	US population, 1,368 subjects	[46]
				No significant association with SSc and -related internal organ involvement	European Caucasian population, 977 cases/558 controls	[45]
56	rs4769058	30,759,294	T > C	No significant Association with Sickle Cell Diseases	US population, 1,518 and 211 subjects	[120]
57	rs9508835	30,760,561	C > A	**Association with increased risk of acute coronary syndrome (ACS)**	**Non-Hispanic Caucasian population, 19 cases/27 controls**	[121]
58	rs9315050	30,761,908	A > G,T	**Association with asthma**	**Caucasian population, 341 families**	[85]
				No association of HapB with IS	Meta-analysis of 4 studies of 3 Caucasian and 1 Asian population	[38]
				No significant association with CI	Chinese Han population, 236 cases/219 controls	[44]
				No association with IS	Northeastern Chinese Han population, 456 cases/452 controls	[81]
				**Significant association of HapB** (but not rs9315050 as single SNP) **with CAD**	**Meta-analysis of 13 studies of 11 Caucasian and 2 Asian populations,** **4,619 cases in total/4,313 controls in total for HapB** 7,125 cases in total/5,353 controls in total for rs9315050	[48]
				**Significant association of HapB with risk of premature CAD**	**European-American population, 500 cases/500 controls**	[52]
				**Association of HapB with increased risk of MI**	**German population, 1,211 cases/1,015 controls**	[49]
				No association of HapB with early EOCAD or MI	Caucasian and American population, 656 cases/405 controls	[54]
				No significant associations with SSC or -related internal organ involvement	European Caucasian population, 977 cases/558 controls	[45]
				No significant association with xanthomas in hypercholesterolemia	Dutch population, 541 cases/494 controls	[47]
				No association with depression	US population, 1,368 subjects	[46]
59	rs9315051	30,762,040	A > G	**Association with atherosclerotic plaque as measure of cardiovascular disease in HIV patients**	**Spanish Caucasian population, 83 cases/130 controls**	[70]
				No significant association with chronic rhinosinusitis	Canadian (~ 90 % White) population, 206 cases/200 controls	[59]
60	rs3935644	30,763,503	G > A,C,T	No association with cardiovascular disease measured by carotid intima media thickness (cIMT) and atherosclerotic plaque presence in HIV patients	Spanish Caucasian population, 83 cases/130 controls	[70]
61	rs4769060	30,763,740	A > G,T	**Association of AA genotype with greater cellular exposure to gemcitabine**	**Japanese population, 256 subjects**	[122]
				**Significant association with IS in female patients**	**Chinese Han population, 459 cases/462 controls**	[76]
				No significant association with CI	Chinese population, 292 cases/259 controls	[26]
				No significant association with IS	Chinese population, 396 cases/378 controls	[82]
				No association with stroke	Taiwanese population, 291 cases/278 controls	[53]
				No significant association with atherosclerosis	Caucasian population, 828 cases/170 controls	[90]
				No association with cardiovascular disease measured by carotid intima media thickness (cIMT) and atherosclerotic plaque presence in HIV patients	Spanish Caucasian population, 83 cases/130 controls	[70]
62	rs41323349	30,764,017	T > C	No significant association with atherosclerosis	Icelandic population, 713 subjects	[94]
				No association with CAD	US population, 1,552 cases/1,583 controls	[84]
63	rs1132340	30,764,325	A > G	**Association of G allele with decreased risk of T2DM**	**Elderly Greek population, 716 cases/569 controls**	[100]
				No association with CAD	US population, 1,552 cases/1,583 controls	[84]
64	rs9315053	30,764,963	T > G	**Association of regular NSAID users with variant genotype with additional decreased risk of rectal cancer**	**US population (Minnesota), 485 cases/578 controls**	[111]
				No significant association with risk of colon and rectal cancer stratified by fat and fatty acid intake	US population (Utah, Northern California, Minnesota), 1,543 cases/1,900 controls (Colon) and 712 cases/912 controls (Rectum)	[108]
65	rs17239025	30,765,768	G > C	**Significant association with rectal cancer risk and association of NSAID users with wildtype genotype with decreased risk of colon cancer**	**US population (Minnesota), 485 cases/578 controls**	[111]
66	rs17222842	30,765,980	G > A	**Significant negative association with MI**	**Danish population, 2,876 cases/2,858 controls**	[43]
				**Significant association of HapB with CAD**	**Meta-analysis of 13 studies of 11 Caucasian and 2 Asian populations,** **4,619 cases in total/4,313 controls in total**	[48]
				**Significant association of HapB with risk of premature CAD**	**European-American population, 500 cases/500 controls**	[52]
				**Association of HapB with increased risk of MI**	**German population, 1,211 cases/1,015 controls**	[49]
				No association of HapB with early EOCAD or MI	Caucasian and American population, 656 cases/405 controls	[54]
				**Significant association of A allele with protection against xanthomas in hypercholesterolemia compared to GG genotype**	**Dutch population, 541 cases/494 controls**	[47]
				No association with stroke	Taiwanese population, 291 cases/278 controls	[53]
				No association of HapB with IS	Meta-analysis of 4 studies of 3 Caucasian and 1 Asian population	[38]
				No association with depression	US population, 1,368 subjects	[46]

SNPs are listed with number, reference SNP Cluster ID, nucleotide position on chromosome 13 (nt), alleles with reference allele > variant allele and linked studies including analyzed association and study subjects. Associations are marked in bold.

*IS* Ischemic stroke, *CI* Cerebral infarction, *(EO)CAD* (E arly onset) Coronary artery disease, *MI* Myocardial infarction, *T2DM* Type 2 diabetes mellitus, *SSc* Scleroderma, *ISR* In-stent restenosis, *AD* Alzheimer disease, *Hap* haplotype

**Fig. 2:**
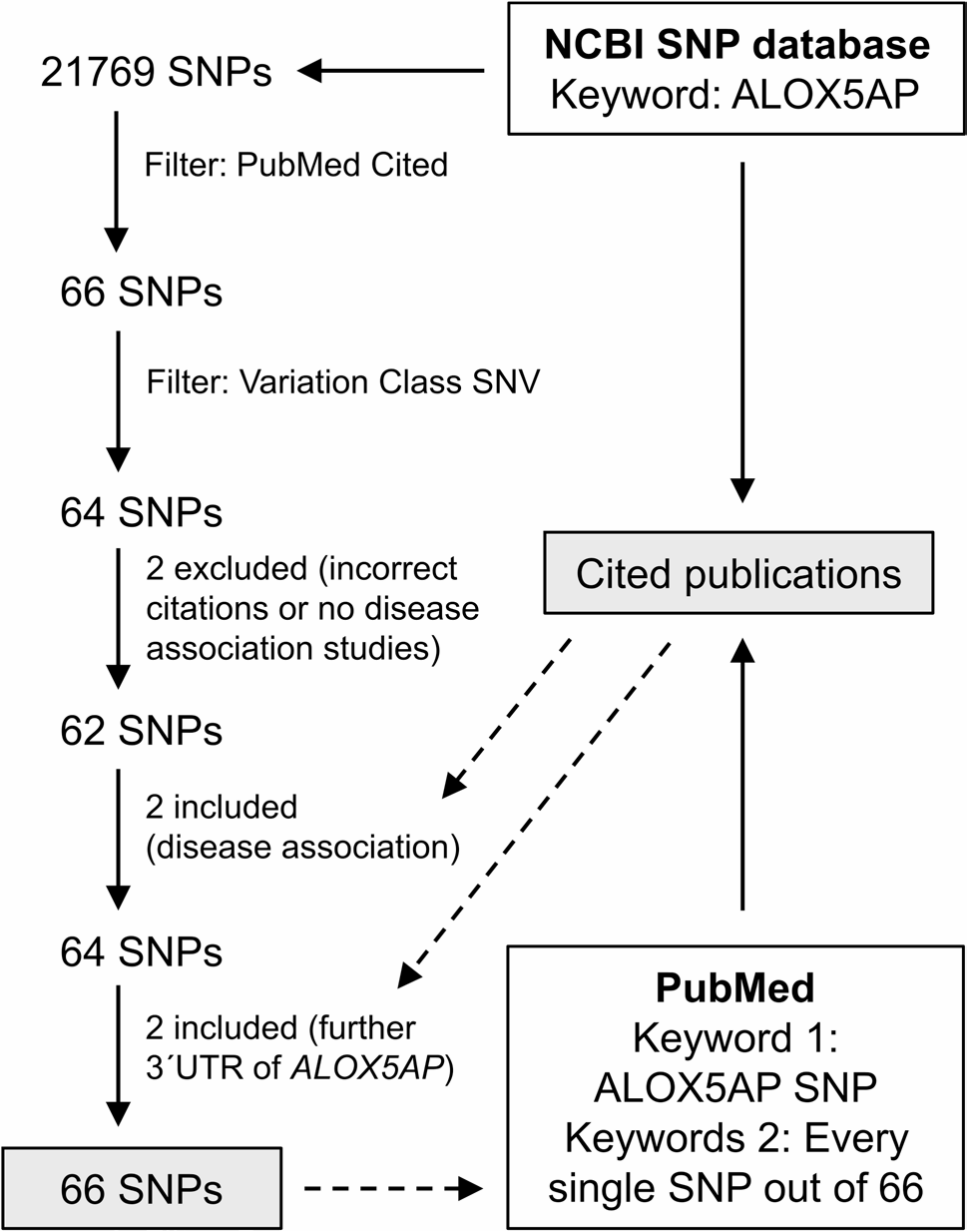
SNP and study selection flowchart

This review is based on a literature search of the NCBI SNP database using the keyword 'ALOX5AP', which initially yielded 21,769 SNPs (Fig. 2). After applying the filters “PubMed Cited” and “Variation Class SNV”, the number of SNPs was reduced to 64. Two of these were excluded due to incorrect citations or a lack of studies investigating disease associations. Based on the literature cited in the publications associated with the remaining SNPs, four additional SNPs were included in this review. Two of these SNPs were selected because they were associated with diseases, but lacked linked citations in the NCBI SNP database. The other two were identified in the literature as being located in the 3´UTR of *ALOX5AP* and could not be retrieved using the keyword 'ALOX5AP' in the NCBI database. Alongside the NCBI SNP database, PubMed was searched directly for additional information. The search terms 'ALOX5AP and SNP' were used in this instance, as well as each of the 66 SNPs, to include further publications with disease associations. The selection of publications was not limited by publication date. The cited studies focused on analysing SNPs associated with various diseases in previous studies, as well as those forming haplotypes (inherited gene combinations) in *ALOX5AP*. These variants were often genotyped using the TaqMan genotype assay in case–control studies. The possibility that other SNPs, not examined in these studies, may be associated with the disease under investigation cannot be excluded. In a few studies, the entire gene was sequenced by next-generation sequencing, with certain discovered (rare) SNPs then being genotyped [93]. Further analysis of SNP associations revealed that the main challenges in many cases were the small study size and restriction to a specific population. Most of the 66 SNPs are found in non-coding regions, while two SNPs (rs41351946 (38) and rs41323349 (62)) are located in exons of *ALOX5AP*. Both are missense variants, leading to an alternative amino acid in FLAP. As previously mentioned, in addition to the study of individual SNPs, the occurrence of haplotypes, a set of polymorphism alleles that co-occur on a chromosome, is also discussed in order to examine allele variation on a larger scale [32]. In *ALOX5AP* the haplotypes HapA (rs17222814 (13), rs10507391 (27), rs4769874 (49), rs9551963 (55)) and HapB (rs17216473 (16), rs10507391 (27), rs9315050 (58), rs17222842 (66)), each consisting of four SNPs, have been increasingly studied.

## Diseases associated with SNPs in *ALOX5AP*

The 66 selected *ALOX5AP* SNPs were studied in the context of several diseases. In some cases, a given SNP has been linked to a particular disease by only one study, whereas others have been investigated across multiple studies addressing different diseases. The main diseases associated are cardiovascular diseases such as coronary artery disease (CAD), myocardial infarction (MI), and ischemic stroke (IS), as well as various types of cancer. As these diseases are the leading causes of death worldwide, tremendous research is underway to identify the genetic variants that influence their risk [33, 34]. Additionally, individual studies have focused on other diseases that are common in the global population, such as asthma, type 2 diabetes mellitus (T2DM) and Alzheimer's disease (AD). The role of inflammatory processes in the pathogenesis of CAD, taking place in the vessel wall and within atherosclerotic plaques, is well recognized, drawing attention towards the leukotriene pathway including FLAP [24, 25]. CAD is a cardiovascular disease caused by atherosclerosis or atherosclerotic occlusion of the coronary arteries [35]. This can result in an inadequate supply of blood and oxygen to the heart muscle, which can lead to the most serious complication of CAD, the MI [24, 36]. IS, also known as cerebral infarction (CI), is a complex multifactorial disorder caused by the sudden loss of blood supply to an area of the brain [37]. The genetic influence on the risk and prognosis of IS has been demonstrated in twin, family and animal model studies [38]. Inflammation-mediated destabilization and rupture of atherosclerotic lesions are thought to be the main causes of the development of IS, suggesting a possible role for FLAP in the development of the disease [26]. While a significant association of several *ALOX5AP* SNPs with IS was found in Central European or Chinese patients, studies in North Americans failed to prove these results. This divergence may be due to genetic differences between populations of different ancestries, and has also been observed in the association of SNPs with other diseases [25]. This review aims to provide an overview of studies that have examined the association between selected *ALOX5AP* SNPs and different disease phenotypes. The following section discusses selected SNPs which were discussed in several studies, yielding diverse and sometimes contradictory results.

## Selected SNPs in *ALOX5AP*

### rs17222814 (13)

SNP number 13 with the Reference SNP cluster ID rs17222814 is mapped at nucleotide position 30,725,416 on chromosome 13, which is located in the upstream region of NM_001629.4 (transcript variant 1) and in intron 1 of NM_001204406.2 (transcript variant 2) of *ALOX5AP*. Nucleotide base G is the reference allele, and A is the variant allele. Rs17222814 (13) is a part of the HapA haplotype, and its occurrence has been studied in relation to several diseases. Most of the studies were unable to establish a link with any of the investigated diseases, including CAD, IS, MI, scleroderma (SSc), depression and xanthomas in hypercholesterolemia [38–53]. For early onset CAD (EOCAD), which describes the occurrence of CAD in younger adults, one study found an association with HapA in a Caucasian cohort with an age of 55 years or less, but no association was found in Americans or the overall sample population [54]. Compared to the negative results for CAD, the association between rs17222814 (13) and EOCAD could be due to the younger age of the cohort studied, especially since both studies used Caucasian and American populations, excluding the effect of demographic genetic differences [39, 54]. In addition, rs17222814 (13) was related to MI and protection against in-stent-restenosis (ISR), which is characterized by progressive narrowing of a coronary lesion previously treated with a stent, for example, in the case of CAD [48, 55].

### rs17216473 (16)

The SNP rs17216473 (16) leads to the variant alleles A and T at nucleotide position 30,729,828 in comparison to the reference allele G. This position is part of the upstream region of NM_001629.4 (transcript variant 1) and intron 1 of NM_001204406.2 (transcript variant 2) of *ALOX5AP*. Rs17216473 (16) was studied as a single SNP in relation to several diseases and also in the context of haplotype occurrence, as it belongs to the HapB haplotype. In the cited studies, no association with EOCAD, IS, depression, SSc and SSc-related interstitial lung disease and xanthomas in hypercholesterolemia was observed [38, 45–47, 54]. However, a significant association with MI was found by Pavlenko et al. in a Ukrainian population, who revealed that the A allele of rs17216473 (16) was associated with an increased risk of MI, whereas the G allele was associated with the absence of MI. All homozygotes with the A allele (AA genotype) in this study suffered from MI. The GG genotype was significantly associated with an absence of MI, whereas the GA genotype was not significantly associated with MI [56]. In this study, a subject group of 95 cases and 110 controls were included. By studying the association of the haplotypes, Crosslin et al*.* found no association between the HapB haplotype with MI in a Caucasian and American population [54], whereas this association was observed in a German population [49]. Moreover, HapB was significantly associated with (premature) CAD in a meta-analysis and a case–control study of a European-American population [48, 52]. Regarding ISR, two studies revealed contrary results. Pleva et al*.* found no association of rs17216473 (16) with ISR, while Shah et al*.* showed an increased risk of ISR [42, 57]. The studies were performed on one American and one European population, which may produce conflicting results, given that different populations exhibit different SNP occurrences [58]. In addition, the relatively small sample sizes of 46 and 160 cases used in both studies, respectively, may also lead to less stable results.

### rs4073259 (18)

SNP number 18 rs4073259 is located at 30,732,134 nt on chromosome 13, upstream of NM_001629.4 (transcript variant 1) and in intron 1 of NM_001204406.2 (transcript variant 2) of *ALOX5AP* and results in the variant allele A while G is the reference allele. No significant association was observed for rs4073259 (18) with MI, thyroid, breast and prostate cancer risk [43, 59–61]. For CI, two studies found that the A allele was significantly associated with the development of the disease, while the frequency of the G allele and GG genotype was significantly lower in cases of IS [44, 62]. Those with the GG genotype were 0.57 times less likely to develop CI than those with the AA genotype [44]. Of note, these results were not confirmed by two other studies, which found no association with IS [38, 63]. Differences between the results of different studies may be due to differences in sample size, SNP detection methods, statistical tests and inclusion criteria for case and control groups [64]. The study that found an association with IS included 236 cases and 219 controls. In contrast, the other studies consisted of a larger number of subjects with twice the population size and a meta-analysis of four studies, which is beneficial because it considers the limitations of individual studies in terms of study size [65].

### rs17222919 (20)

According to the previously described SNPs, rs17222919 (20) is also part of the upstream region of NM_001629.4 (transcript variant 1) and intron 1 of NM_001204406.2 (transcript variant 2) of *ALOX5AP* at nucleotide position 30,734,192. The variant alleles A or G replace the reference allele T. Rs17222919 (20) has been included in several studies focusing on IS, with five studies showing a significant association with IS [37, 64, 66–68]. Fan et al*.* and Yang et al*.* both analyzed Chinese populations for the risk of IS and found that the G allele frequency was significantly lower in the IS group [37, 67]. In contrast, in an Iranian population, the presence of the G allele was shown to increase the risk of IS, while the presence of the T allele decreased the risk of developing IS [64]. In another Chinese population, the TG genotype was significantly higher in IS cases [68]. The occurrence of SNPs can vary between different populations due to the different natural selection pressures [58]. In addition, other parts of the study design are also critical, such as sample size, inclusion criteria for subjects, and how results are interpreted. These can lead to different results [64]. The same applies to meta-analyses, where the methods used must be just as rigorous as for any other study. As reviews of previous research studies, meta-analyses can provide a more accurate estimate of the risk factors for diseases or other outcomes than any single study contributing to the pooled analysis. However, they are not foolproof tools. There are several examples of meta-analyses of the same question coming to opposite conclusions [65]. This is also evident in this case of the SNP rs17222919 (20), where a meta-analysis of four different Asian studies found no significant association with IS in the overall population, while Ye et al*.* suggested an association in another meta-analysis in the overall population of five Chinese and Korean populations [38, 66]. Differences in literature search, inclusion or exclusion criteria for individual studies, heterogeneity, and statistical analysis can all lead to different meta-analysis results. Heterogeneity of included studies is sometimes assessed by sensitivity analysis. This is used to examine the effect of studies that are abnormal in terms of conduct or results, or that have a significant impact on the analysis [65]. The sensitivity analysis in the meta-analysis by Ye et al*.* showed instability in the results, which may affect the comparability with other meta-analyses [66].

### rs9579646 (23)

The SNP rs9579646 (23) leads to the variant alleles A, C and T at nucleotide position 30,736,442 in comparison to the reference allele G. This position is part of intron 1 of NM_001629.4 (transcript variant 1) and intron 2 of NM_001204406.2 (transcript variant 2) of *ALOX5AP.* Rs9579646 (23) was found to be significantly associated with IS in a US population [69]. In three Chinese Han populations, rs9579646 (23) was also associated with IS. Zhang et al*.* and Qu et al*.* found that the GG genotype or G allele was associated with an increased risk of CI, and Sun et al*.* revealed that the AG genotype was associated with a reduced risk of stroke compared with the AA genotype. These results could not be confirmed by two other studies that found no significant association with IS in Chinese populations [37, 68]. As discussed above, differences in the results of different studies may be due to a deviating study design, including SNP detection, subject inclusion criteria, and evaluation of the data obtained [64]. The SNP rs9579646 (23) was also studied in relation to cardiovascular disease, for which Merhi et al*.* found a significant association with a reduced risk of CAD [25]. Cardiovascular disease can be a cause of death in HIV patients. In a study of HIV patients, no association of rs9579646 (23) with cardiovascular disease was found, which is consistent with the findings of Merhi et al*.* [70].

### rs10507391 (27)

The SNP rs10507391 (27) is located at nucleotide position 30,737,959 and is part of intron 1 of NM_001629.4 (transcript variant 1) and intron 2 of NM_001204406.2 (transcript variant 2) of *ALOX5AP*. This SNP results in the appearance of the variant alleles C and T compared to the reference allele A and is part of both the HapA and HapB haplotypes. Several studies have been carried out on this SNP, both as a single SNP and in haplotypes associated with different diseases. Many of the cited studies focused on IS, leading to different results. While most studies, including two meta-analyses, found no significant association with IS [38, 44, 50, 53, 71–76], Li et al*.* revealed a significant association with IS in Caucasians from Europe, but not from other subject groups [77]. This is consistent with the findings of Domingues-Montanari et al*.*, who found, that the T allele of rs10507391 (27) was significantly associated with an increased risk of IS [78]. Yao et al. demonstrated an association of the TT/TA genotype and an increased risk of CI in a Chinese population, compared to the AA genotype [79], and in another Chinese population, the AA genotype and A allele were associated with atherothrombotic stroke, an important subtype of IS [51]. The different results indicate diverging study designs, and also a high variability in the occurrence of SNPs across different populations. Zheng et al*.* took this into account in their meta-analysis, which involved analyzing the overall Caucasian and Asian populations and finding no association with IS [38]. In addition to analyzing the occurrence of rs10507391 (27) alone, this SNP was also studied in combination with other SNPs. The presence of both rs10507391(27) and rs12429692 (28) was found to significantly reduce the risk of CI [80]. The opposite effect was found for the haplotype composed of rs9579646 (23) and rs10507391 (27) (GT and GA), which was shown to be significantly associated with an increased risk of IS [81]. The same was true for the interaction between rs10507391 (27) and rs776746 of cytochrome P-450 3A5 (CYP3A5) (GG genotype) or rs20417 (COX-2) [26, 82, 83].

Regarding CAD, Assimes et al*.* and Hartiala et al*.* found no association of rs10507391 (27) or HapA haplotype in either African Americans or Caucasians [39, 84], while a meta-analysis and a case–control study revealed a significant association of HapB and (premature) CAD in the overall population or a European-American population [48, 52]. Moreover, Crosslin et al*.* detected an association of HapA with EOCAD in Caucasians under 55 years old [54]. The HapA and HapB haplotypes were also associated with MI [48, 49]. Moreover, it was also found that rs10507391 (27) is associated with an increased risk of ISR, while the HapA haplotype should be protective against restenosis [42]. Some of the cited studies focused on the associations between rs10507391 (27) and lung function, showing the A allele as a risk factor of asthma [85, 86]. Contrasting results were obtained by Via et al*.* for the co-occurrence of the A allele with rs17525488 of the leukotriene A_4_ hydrolase (LTA_4_H) gene, which was associated with a reduced risk of asthma and lower baseline lung function [87]. In addition, the combination of rs10507391 (27) with two other genomic variations of the LTA_4_H gene rs2540491 and rs2540487 was found to increase bronchodilator responsiveness with leukotriene modifiers [88].

Further associations were made between rs10507391 (27) and SSc and SSc-related interstitial lung disease, as well as decreased risk of myeloid leukemia for the AA genotype [45, 89]. No significant association of rs10507391 (27) was found for atherosclerosis, depression, AD and xanthomas in hypercholesterolaemia [24, 43, 46, 47, 90, 91].

### rs4769874 (49)

Rs4769874 (49) is located at 30,752,304 nt on chromosome 13 and belongs to intron 3 of NM_001629.4 (transcript variant 1) and intron 4 of NM_001204406.2 (transcript variant 2). The reference allele G is replaced by the variant allele A, and rs4769874 (49) is also part of the HapA haplotype of *ALOX5AP*. The A allele of rs4769874 (49) was found to be significantly associated with an increased risk of AD in a Czech population (1.41-fold) [91]. Studies examining the association with IS have produced conflicting findings. Kaushal et al*.* identified a significant association of rs4769874 (49) with IS among individuals of European ancestry from a US population in Greater Cincinnati/Northern Kentucky, including 357 cases and 482 controls [69]. This finding was not confirmed by five Asian studies that found no significant association of rs4769874 (49) with IS [26, 53, 76, 81, 82]. The sample sizes of the studies are comparable, which suggests that the differences found are due to differences in the studies’ setup or in the populations’ genetic characteristics. This assumption becomes more likely when considering the meta-analysis by Zheng et al., which included four Caucasian and nine Asian studies. Here, no association of either rs4769874 (49) or HapA haplotype with IS could be detected in the overall population [38]. Three further studies also found no association between the HapA and stroke in a Caribbean Hispanic and a European population or a Chinese population [50, 51, 72].

Regarding CAD, rs4769874 (49) was shown to be significantly associated with an increased risk in a Lebanese population of 1,370 cases and 589 controls [25], while a meta-analysis could not confirm these results [48]. The HapA haplotype did not show an association with CAD [38, 49, 52], but with EOCAD [54] and MI in a meta-analysis [48]. Moreover, there was no association found of rs4769874 (49) with the acute coronary syndrome, depression, myeloid leukemia, SSc, and xanthomas in hypercholesterolemia [43, 45–47, 89, 92].

### rs9551963 (55)

The SNP rs9551963 (55) leads to the variant alleles C and T at nucleotide position 30,758,410 in comparison to the reference allele A. This position is part of intron 3 of NM_001629.4 (transcript variant 1) and intron 4 of NM_001204406.2 (transcript variant 2) of *ALOX5AP*. Rs9551963 (55) was studied as a single SNP and in a wider genetic context, as it belongs to the HapA haplotype. Regarding IS, two studies found a significant association between the AC genotype or the C allele and increased risk of IS in hypertensive patients or female patients [44, 76], while the others could not confirm these findings [26, 38, 51, 53, 72, 74, 75, 81, 82]. In combination with smoking, Yao et al. found an association of the CC/CA genotype and an increased risk of CI compared to the AA genotype [79].

In terms of cardiovascular disease, the HapA haplotype, including rs9551963 (55), was associated with EOCAD and MI (only in a meta-analysis) but not CAD and IS, as previously discussed for rs17222814 (13) [39, 48–50, 52, 54]. Additionally, rs9551963 (55) could also not be identified as a single SNP associated with CAD in a meta-analysis [48]. However, rs9551963 (55) was found to be associated with a reduced likelihood of MI in a Danish population of 2,876 cases and 2,858 controls. The results were comparable between the sexes, but were significant only in men [43].

The rs9551963 (55) variant was also studied in relation to lung function and was found to be associated with an increased response to bronchodilators when used in combination with leukotriene modifiers, provided that the rs2540491 or rs2540487 variants of the LTA_4_H gene are also present [88]. An association with asthma was not found [87].

In a Dutch population of 541 cases and 494 controls, the A allele of rs9551963 (55) was significantly associated with an increased risk of xanthomas in hypercholesterolemia compared with the CC genotype [47]. No other associations were identified between rs9551963 (55) and atherosclerosis, depression and SSc, and SSc-related internal organ involvement [41, 45, 46].

### rs41351946 (38) and rs41323349 (62) variants within the exon region of *ALOX5AP*

**Fig. 3:**
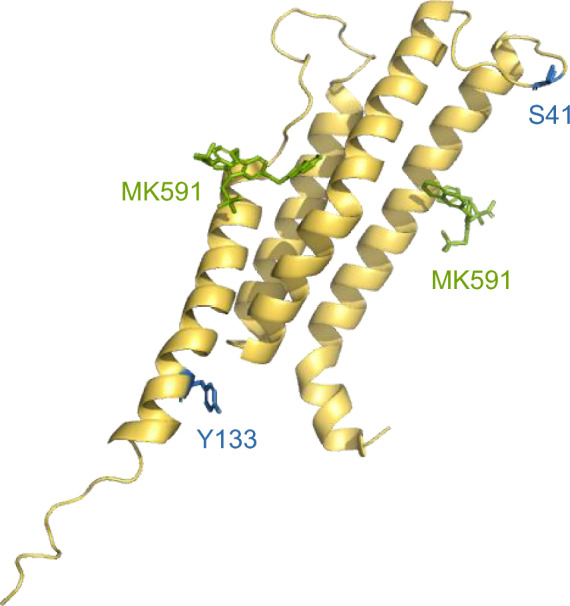
Cartoon rendering of the FLAP monomer. The amino acid positions S41 and Y133 of NP_001620.2 (isoform 1) which are changed by the missense SNPs rs41351946 (38) and rs41323349 (62) are marked in blue. MK591 is shown as stick rendering (green).

Among the more than 20,000 SNP variants reported for *ALOX5AP*, 259 SNPs are missense variants, located within exons and result in amino acid replacement of FLAP. Of these 259 missense SNPs, only rs41351946 (38) and rs41323349 (62) were investigated according to disease association generating a PubMed citation (Fig. 3).

Rs41351946 (38) at nucleotide position 30,744,112 on chromosome 13 leads to the variant alleles G and T, where C is the reference allele. This position is located in exon 2 of NM_001629.4 (transcript variant 1) and exon 3 of NM_001204406.2 (transcript variant 2), changing the amino acid serine (Ser) to arginine (Arg) of FLAP at 41 aa of NP_001620.2 (isoform 1) and 98 aa of NP_001191335.1 (isoform 2). Recent site-directed mutagenesis studies on FLAP investigated the role of Ser41 (isoform 1) in 5-LOX/FLAP complex formation and leukotriene biosynthesis. Replacement of Ser41 by alanine (Ala) or aspartate (Asp) did not alter lipid mediator production or the interaction between 5-LOX and FLAP [17]. For the SNP rs41351946 (38), only one study was cited, in which Postula et al*.* found an association with T2DM in a Polish population of 303 subjects [93].

Rs41323349 (62) is located at nucleotide position 30,764,017 on chromosome 13 and belongs to exon 5 of NM_001629.4 (transcript variant 1) and exon 6 of NM_001204406.2 (transcript variant 2). The SNP consists of the variant allele C, where T is the reference allele, and results in an amino acid change from tyrosine (Tyr) to histidine (His) of FLAP at position 133 aa of NP_001620.2 (isoform 1) and 190 aa of NP_001191335.1 (isoform 2). Only two studies are linked to rs41323349 (62), revealing no association with atherosclerosis and CAD [84, 94].

## Conclusion

SNPs are the most common type of DNA variation and are shared by a population with at least 1% occurrence. It has been shown that SNPs contribute to the development of various diseases and have therefore been the basis of a large number of association studies in recent years [29]. In this review, we focused on SNPs of the *ALOX5AP* gene encoding FLAP, which were studied for the association with various diseases. As FLAP is important in the leukotriene biosynthesis as a helper protein for 5-LOX, especially diseases associated with inflammatory processes such as CAD, MI and IS were part of a large proportion of the studies [95]. However, this does not exclude an alternative action beyond the role in lipid mediator formation. There is a large discrepancy between the results obtained, as some studies have suggested an association of several SNPs, including rs4073259 (18), rs17222919 (20), rs10507391 (27), rs4769874 (49) and rs9551963 (55) with IS, CAD or MI, but others have not confirmed this. The study design, including sample size, inclusion and exclusion criteria for cases and controls, SNP detection methods, and statistical analysis is essential and may lead to different outcomes of the studies [64]. In addition, SNPs have different frequencies in different populations due to the varying natural selection pressures experienced by different populations. Consequently, drawing a generalized conclusion is challenging. By including diverse populations and larger sample sizes, meta-analyses allow the results of different association studies to be contextualized within a broader framework. However, meta-analyses are also dependent on the study design and cannot provide a definitive understanding of the association of different SNPs with different diseases [96]. Most of the studies cited in this review that investigated *ALOX5AP* polymorphisms and their associations were conducted between the 2000s and 2015, at a time when gene association studies were prevalent. Over the last 5–7 years, the focus has shifted towards understanding the functional and mechanistic roles of its protein product, FLAP. This is perhaps partly due to inconsistent results, as can be seen in this overview. These studies examine the impact of altered FLAP expression on inflammatory signaling, immune cell regulation and lipid mediator synthesis in various disease contexts, including glioma, osteosarcoma and stress-induced organ injury [97–99]. This shift highlights the fact that disease relevance may arise from altered expression and signaling rather than from genetic variation alone. Therefore, future studies should combine the identification of *ALOX5AP* SNPs with functional genomic approaches and clinical data to further complement our understanding of FLAP's role in disease processes, helping to identify individuals at increased risk of disease and initiate targeted therapies. If certain *ALOX5AP* SNPs are consistently linked to specific diseases, they could enable personalized medicine approaches by identifying high-risk individuals and guiding the use of FLAP-targeted or leukotriene-modulating therapies. Such findings would also inform the development of more precise and effective drugs that specifically address the molecular mechanisms influenced by these genetic variants.

## Data Availability

No datasets were generated within this study.

## Abbreviations

FLAP: 5-Lipoxygenase-activating protein
SNP: Single-nucleotide polymorphism
COX: Cyclooxygenase,
LOX: Lipoxygenase
LT: Leukotrienes
AA: Arachidonic acid
5-HPETE: 5-Hydroperoxyeicosatetraenoic acid
IS: Ischemic stroke
CI: Cerebral infarction
(EO)CAD: (Early onset) Coronary artery disease
MI: Myocardial infarction
T2DM: Type 2 Diabetes mellitus
SSc: Scleroderma
ISR: In-stent restenosis
AD: Alzheimer disease
Hap: Haplotype
